# Early presymptomatic cholinergic dysfunction in a murine model of amyotrophic lateral sclerosis

**DOI:** 10.1002/brb3.141

**Published:** 2013-05-13

**Authors:** Caty Casas, Mireia Herrando-Grabulosa, Raquel Manzano, Renzo Mancuso, Rosario Osta, Xavier Navarro

*Brain and Behavior* 2013; **3**(2): 145–158

On page 149, the sentence “In these cisterns the sigma-1 receptor is present to buffer calcium entry overload (Mavlyutov et al. 2010)” should be changed to the following: “At these cisterns, the sigma-1 receptor may play an indirect action modulating intracellular calcium release (Mavlyutov et al. 2010).”

We regret this misinterpretation. It has no consequences for the reported analyses and conclusions.
